# Investigating the mediating role of comprehensive self-efficacy in the relationship between altruistic factors and voluntary non-remunerated blood donation behavior

**DOI:** 10.3389/fpubh.2026.1795974

**Published:** 2026-04-10

**Authors:** Xiaogai Han, Shicheng Zhang, Xinhao Zhang, Yujing Liu, Shichang Yang, Xuefang Kou, Tao Wen, Shuqin Zhang, Lu Qiao, Yuxiang Chen, Li Zhang, Yongfei Zhou, Wenan Dong

**Affiliations:** 1 Henan Red Cross Blood Center, Zhengzhou, China; 2 Institute of Hemu Biotechnology, Beijing Hemu Biotechnology Co., Ltd., Beijing, China; 3 Henan Medical University, Xinxiang, China

**Keywords:** altruistic factors, cross-sectional study, mediating effect, self-efficacy, voluntary non-remunerated blood donation

## Abstract

**Objective:**

To investigate the relationship between altruistic factors and voluntary non-remunerated blood donation behavior, and to examine the potential mediating role of comprehensive self-efficacy in this relationship.

**Methods:**

A cross-sectional survey was conducted from June 2023 to April 2024 in Zhengzhou and Xinxiang, Henan Province, China. Using stratified random sampling, 9,622 residents aged 18–60 years were recruited. Data were collected via questionnaires assessing sociodemographic characteristics, altruistic factors (score range: 0–3), comprehensive self-efficacy regarding blood donation (a composite score of knowledge, situational confidence, policy awareness, and service evaluation), and self-reported blood donation history. Blood donation behavior was categorized for analysis as non-donor versus donor (≥1 donation). Multinomial logistic regression and bootstrap mediation analysis were employed.

**Results:**

Altruistic factors showed a significant positive association with donation behavior, exhibiting a clear dose–response relationship (ORs ranged from 1.673 to 2.592, all *p* < 0.001). Comprehensive self-efficacy was a strong independent predictor of donation (*p* < 0.001). Crucially, mediation analysis revealed a statistical pattern consistent with full mediation: the association between altruistic factors and donation behavior was significantly mediated by comprehensive self-efficacy (indirect effect: 0.335, 95% CI [0.301, 0.372]), with no significant direct effect detected. Owing to the cross-sectional nature of this study, this finding only describes the statistical association between variables, and cannot confirm a definitive causal mediating pathway. Significant demographic disparities were also observed.

**Conclusion:**

Altruistic motivation provides the foundational impetus for blood donation, but its translation into action is channeled through an individual’s perceived comprehensive self-efficacy. This suggests that promotion strategies should evolve from solely emphasizing altruism towards integrated efforts that systematically build public knowledge, confidence, and trust in the donation process. Integrated strategies that enhance both altruistic motivation and comprehensive self-efficacy are essential for improving donor recruitment and retention.

## 1 Introduction

Blood is an indispensable medical resource with ever-growing clinical demand (1). In the absence of artificial substitutes, the blood supply relies entirely on voluntary non-remunerated donations from healthy individuals (2). In China, continuous promotion of voluntary donation has led to sustained growth in donor numbers and collection volumes over the past two decades, placing the country among the global leaders in this regard. Nevertheless, the national blood supply remains in a state of precarious equilibrium. This is characterized by marked regional disparities, as well as persistent seasonal and structural shortages (3).

Public knowledge of donation policies are critical factors influencing donation willingness and behavior (4). Common barriers include insufficient awareness, misconceptions about health effects, and misunderstandings regarding reimbursement policies (5, 6). Among various motivations for donation, altruism is widely regarded as the most fundamental and robust driver (7). Extensive evidence consistently identifies the desire to help others as a primary motivator, especially among repeat donors (8, 9). However, a clear gap often exists between intention and actual behavior; strong altruistic motivation does not invariably translate into donation, suggesting the involvement of other psychological factors.

Self-efficacy, defined as an individual’s confidence in their ability to successfully perform a specific behavior, is considered a key construct in social cognitive theory and may serve as a potential link between motivation and action (10). In the context of blood donation, self-efficacy reflects one’s confidence in being able to complete the donation process. Evidence suggests that self-efficacy is associated with donation intention and may help reduce donation-related fears, thereby supporting both first-time and repeat donations (11–14). Therefore, translating prosocial motivation into actual behavior may depend both on altruistic drive and on an individual’s perceived capability to succeed.

In this study, altruistic factors specifically refer to the altruistic motivations driving individuals to participate in voluntary blood donation, including three core dimensions: pure altruism (donating love and helping others), self-actualization-oriented altruism (achieving self-worth through prosocial behaviors), and reciprocal altruism (repaying society for having received blood donation assistance). These factors constitute the intrinsic prosocial motivation that prompts individuals to form the intention to donate blood. Blood donation behavior in this study is defined as voluntary non-remunerated blood donation among eligible individuals aged 18 to 60 years, which is further categorized into non-donation, first-time donation, and repeat donation based on the actual number of donations. It is a concrete external manifestation of prosocial behaviors driven by psychological factors such as altruistic motivation.

Although previous studies have separately examined the roles of knowledge, altruism, or self-efficacy in donation behavior, how these factors interact remains inadequately understood (4, 15, 16). In particular, whether altruistic factors are linked to donation behavior directly, or indirectly through enhancing self-efficacy, constitutes a salient research gap. Clarifying this potential mediating pathway would deepen the theoretical understanding of the psychological process underlying donation behavior and inform more precisely targeted intervention strategies. Notably, although previous studies have examined the mediating role of self-efficacy in blood donation among Western or small-scale samples, limited evidence is available from large-scale Chinese populations (16).

Therefore, based on the core connotations of social behavior theory and the self-efficacy theory, and combined with the prosocial nature of voluntary blood donation, this study constructs an integrated mediating theoretical framework. The social behavior theory posits that an individual’s prosocial behavior is not merely driven by intrinsic motivation, but by the combined effect of motivation and the individual’s perception of behavioral competence (17). As the core intrinsic motivation for voluntary blood donation, the transformation of altruistic motivation into actual behavior thus relies on key mediating variables to connect motivation and behavior. This theoretical proposition creates a theoretical space for the introduction of self-efficacy theory, which clarifies that an individual’s perception of their ability to successfully perform a specific behavior serves as an important bridge linking intrinsic motivation and external behavior (18). In the context of voluntary blood donation, comprehensive self-efficacy reflects an individual’s comprehensive perception of their ability to complete the blood donation process, which can precisely act as a key mediator in the transformation of altruistic factors into blood donation behavior. Based on this, comprehensive self-efficacy is set as the mediating variable between altruistic factors and blood donation behavior in this study. Through a large-scale survey of nearly 10,000 residents in Henan Province, we aim to examine: (1) the association between altruistic factors and voluntary non-remunerated blood donation behavior, and (2) the potential mediating role of comprehensive self-efficacy in this relationship. The findings are expected to provide empirically grounded evidence and targeted theoretical insights for refining donor recruitment and retention strategies from a social-psychological perspective.

## 2 Materials and methods

### 2.1 Study design and participants

This study employed a cross-sectional survey design. From June 2023 to April 2024, participants were recruited using a stratified random sampling method at blood donation centers, universities, and community centers in Zhengzhou and Xinxiang, Henan Province. The study cohort comprised Chinese citizens aged 18 to 60 years. Exclusion criteria included: (1) individuals with mental disorders impairing survey compliance; (2) those with severe physical conditions contraindicating blood donation; and (3) potential participants unable to comprehend questionnaire content due to factors such as illiteracy or insufficient education.

A stratified sampling approach was employed based on the age distribution of previous blood donors in Zhengzhou (18–25 years: 33%; 25–35 years: 24%; 35–45 years: 23%; 45–55 years: 18%; 55–60 years: 2%). With a margin of error *δ* = 0.01 and significance level a = 0.05, the minimum sample size was calculated using the formula where the national average blood donation rate *π* was set at 11%. For a two-tailed test with a significance level of a = 0.05, the critical value is u_a/2=_u_0.025_. Referring to the standard normal distribution table, we find that u_0.025_ = 1.96


$$n=\frac{{u^{2}}_{a/2}\cdot \pi \cdot (1-\pi )}{\delta^{2}}$$

This calculation yielded a minimum required sample size of 3,760 participants. Considering potential sample attrition, incomplete data, and ineligible cases, the target sample size was expanded to approximately 4,000. Accordingly, the stratum-specific sample sizes were determined as follows: 1320 cases aged 18–25 years, 960 cases aged 25–35 years, 920 cases aged 35–45 years, 720 cases aged 45–55 years, and 80 cases aged 55–60 years, respectively. Stratification was first conducted by age, followed by proportional sample size allocation within each age stratum. Subsequently, simple random sampling was adopted to recruit participants at each recruitment site. On-site recruitment was implemented for the study, and all participants signed the informed consent form prior to completing the questionnaire. Within each stratum, data collection was performed via random sampling at voluntary blood donation stations. Ultimately, 9,622 valid questionnaires were collected.

### 2.2 Measures

#### 2.2.1 Demographic and sociological questionnaire

The study utilized a self-developed questionnaire to collect socio-demographic characteristics, including: gender; age; educational attainment; occupation; household registration type (urban vs. rural); and personal and familial history of blood donation and transfusion.

#### 2.2.2 Altruistic factors

Participants’ level of altruistic factors was evaluated using the survey item: “What is your primary motivation for blood donation?” Respondents could select multiple options. Of these, three responses were theoretically defined as core altruistic motivations: (1) “to offer love and help others— representing pure altruism (19),”(2) “to achieve self-actualization— considered an internalized form of altruism fulfilled through prosocial behavior (9),” (3) “to reciprocate society due to having received blood donations from others— aligning with the concept of gratitude or reciprocal altruism (8).” Each selected altruistic option was assigned 1 point. The total altruistic factors score represented the sum of chosen altruistic motivations, ranging from 0 to 3. Higher scores indicated more diverse and stronger altruistically driven donation motivations.

#### 2.2.3 Comprehensive self-efficacy (multidimensional construct)

In line with research that adapts efficacy measures to specific health contexts (20, 21), the term “comprehensive self-efficacy” here refers to a donation-specific, multidimensional construct. For blood donation—a structured healthcare behavior—perceived capability depends not only on personal confidence but also on procedural knowledge, system trust, and environmental comfort. We therefore operationalize it as a composite of four dimensions representing an individual’s holistic readiness to donate: (1) knowledge mastery, (2) situational confidence, (3) policy awareness, and (4) service evaluation.

A composite self-efficacy score was constructed by integrating four dimensions to reflect individuals’ confidence in successfully completing blood donation: (1) Knowledge Mastery: Understanding of blood donation-related knowledge provides the cognitive basis upon which efficacy beliefs are formed. Assessed through understanding of donation knowledge, scored from 0 (“unaware”) to 4 (“highly knowledgeable”); (2) Behavioral Confidence: This dimension was assessed using a situational behavioral preference method, grounded in the theory of situation-specific self-efficacy. An individual’s preference for donation venues reflects a decision-making process that integrates their internal confidence with an assessment of external environmental risks. Selections indicating a preference for fixed, professional donation sites (e.g., blood centers or stations, donation rooms)—characterized by “high controllability and low uncertainty”—were each assigned 1 point. This scoring reflects a confidence strategy whereby individuals seek optimal environments to ensure successful donation. Selections indicating a preference for mobile or temporary blood collection vehicles (e.g., street mobiles, vehicles at workplaces/residential areas)—environments with higher variability—were each assigned 0.5 points, reflecting moderate confidence in navigating settings with greater uncertainty. Critically, selecting “Do not know where to go” resulted in a score of 0 points for this dimension, irrespective of any other concurrent selections. This scoring rule captures a fundamental lack of behavioral confidence stemming from the complete absence of information needed to initiate the action, representing the lowest level of situational control. The scores from all applicable selections were summed to create the dimension score. A higher score indicates stronger comprehensive situational confidence, as evidenced through the individual’s environmental choices; (3) Policy Awareness: Awareness of such incentive policies influences an individual’s expectations regarding behavioral outcomes and is a key component of self-efficacy. Measured by knowledge of free blood reimbursement policies, scored from 0 (“uninformed”) to 2 (“fully informed”); and (4) Service Evaluation: Positive prior experience serves as the most immediate source of self-efficacy. Rated based on satisfaction with donation services, scaled from 1 (“very dissatisfied”) to 5 (“satisfied”). The overall comprehensive self-efficacy score was calculated as the arithmetic mean of these four dimensional scores, with higher values indicating stronger comprehensive self-efficacy.

#### 2.2.4 Cognitive factors assessment

A composite cognitive factor score was constructed by integrating four dimensions to reflect individuals’ awareness, exposure, and informational needs regarding blood donation: (1) Level of Understanding: Assessed through the question “How much do you know about blood donation?” Responses were scored on a 4-point scale: 0 (“unaware”), 1 (“heard of it but know little”), 2 (“know a little”), and 3 (“know quite a bit”). (2) Number of Information Channels: Participants indicated the channels through which they had received blood-donation information (e.g., television, internet, community publicity). The total number of selected channels was summed, ranging from 0 to 9. (3) Frequency of Publicity Exposure: Measured by the question “How often do you encounter blood donation publicity?” Responses were scored as 0 (“never”), 1 (“occasionally”), and 2 (“frequently”). (4) Need for Knowledge: Participants were presented with a list of six blood-donation-related topics (e.g., donation process, eligibility criteria) and asked to select those they wished to learn more about. The total number of selected topics was counted, ranging from 0 to 6. The overall cognitive score was calculated as the sum of these four dimension scores, with higher values indicating greater cognitive engagement with blood donation.

#### 2.2.5 Assessment of blood donation behavior

Donation behavior was assessed through the survey item: “What is your total number of blood donations to date?” Based on World Health Organization recommendations and standard Chinese blood collection agency protocols, participants were categorized into: (1) non-donor (0 donations); (2) first-time donor (1 donation); and (3) repeat donor (≥2 donations).

### 2.3 Data collection and quality control

Data were collected through both online (via QR codes generated on the Wenjuanxing platform) and offline (paper-based questionnaires) channels. All investigators underwent uniform training and administered surveys using standardized instructions. Prior to participation, the study purpose was clearly explained, with emphasis on anonymity and data confidentiality, and informed consent was obtained. Upon questionnaire completion, investigators immediately verified response completeness and promptly requested participants to rectify any omissions or logical inconsistencies (22).

Data entry was performed using EpiData software with predefined logic checks. To ensure accuracy, a dual independent entry protocol was implemented.

#### 2.3.1 Permission to reuse and copyright

Permission must be obtained for use of copyrighted material from other sources (including the web). Please note that it is compulsory to follow figure instructions.

### 2.4 Statistical analysis

Data analysis was performed using IBM SPSS Statistics 27.0. Continuous variables were expressed as mean ± standard deviation, while categorical data were presented as frequencies (percentages). Differences in socio-demographic characteristics across blood donation behavior groups were assessed using chi-square tests (23–25). Subsequently, one-way analysis of variance (ANOVA) was employed to compare differences in the total altruistic factors score and the composite self-efficacy score across the three blood donation behavior groups. To provide a more intuitive presentation of variable relationships, data distributions and association patterns were visualized using GraphPad Prism 10 software. Multinomial logistic regression analysis (with “non-donors” as the reference group) was employed to examine the effects of altruistic factors, comprehensive self-efficacy, and demographic variables on donation behavior (26), with results reported as odds ratios (OR) and their 95% confidence intervals (CI).

For the mediation analysis, given that there were no statistically significant differences in altruistic factor scores between first-time donors and repeat donors, and that Model 4 of the SPSS PROCESS macro (Version 5.0) is only applicable to the analysis of dichotomous outcome variables, blood donation behavior was dichotomized into non-donors (0 times) and donors (≥1 time). The potential mediating role of comprehensive self-efficacy in the relationship between altruistic factors and donation behavior was examined using the PROCESS v5.0 macro (Model 4) for SPSS, applying bootstrap sampling with 5,000 iterations. The mediation effect was considered statistically significant if the 95% confidence interval for the estimated indirect effect did not include zero (27, 28). To facilitate the comparison of effect sizes across independent variables and the visualization of the structural path diagram, this study converted the unstandardized path coefficients obtained from PROCESS into standardized coefficients (*β*). Conventional standardization based on standard deviation was used for the path between continuous variables (X → M), and the common approximation standardization for logistic regression was adopted for the path from the mediator to the binary outcome (M → Y). This transformation allowed direct interpretation of the relative effect sizes of each variable on a unified scale.

Prior to ANOVA and logistic regression, all model assumptions were checked. The data satisfied the assumptions of independence, normality, and homogeneity of variance for ANOVA, as well as independent observations and no multicollinearity for logistic regression. All statistical analyses were two-tailed, with a *p* value < 0.05 considered statistically significant.

## 3 Results

### 3.1 Descriptive statistics of core variables

The distribution of altruistic factors scores across different blood donation behavior groups is presented in Table 1. The overall mean altruistic factors score was 1.33 (SD = 0.84). A strong and significant association was observed between altruistic factors score and donation behavior (χ^2^(6) = 153.02, *p* < 0.001). Examination of column percentages reveals a clear gradient: the proportion of individuals with a score of 0 (no altruistic motive) was highest among non-donors (19.0%) and successively lower among first-time (10.6%) and repeat donors (6.5%). Conversely, the proportion of individuals endorsing multiple altruistic motives (scores of 2 or 3) showed an increasing trend from non-donors (40.3%) to first-time (46.5%) and repeat donors (49.1%). This pattern provides preliminary evidence for the construct validity of the altruistic factors measure and its positive association with donation engagement.

**Table 1:** Distribution of altruistic factors score and its association with blood donation behavior [*n* (%)].

Altruism score	Non-donor (*n* = 7,527)	First-time donor (*n* = 937)	Repeat donor (*n* = 1,158)	Total (*N* = 9,622)	χ^2^	*P* value
Score 0, *n* (%)	1,431 (19.0%)	99 (10.6%)	75 (6.5%)	1,605 (16.7%)	153.02	*P* < 0.001
Score 1, *n* (%)	3,063 (40.7%)	402 (42.9%)	515 (44.5%)	3,980 (41.4%)		
Score 2, *n* (%)	2,451 (32.6%)	358 (38.2%)	485 (41.9%)	3,294 (34.2%)		
Score 3, *n* (%)	582 (7.7%)	78 (8.3%)	83 (7.2%)	743 (7.7%)		

Values are presented as *n* (column %). The altruistic factors score (range 0–3) reflects the number of altruistic motivations endorsed. Blood donation behavior categories: Non-donor (0 donations), First-time donor (1 donation), Repeat donor (≥2 donations). Pearson’s chi-square test was performed to assess the association between altruistic factors score and donation behavior group.

### 3.2 Psychometric properties of the comprehensive self-efficacy measure

Exploratory factor analysis using principal component analysis revealed a Kaiser-Meyer-Olkin (KMO) measure of 0.688 and a significant Bartlett’s test of sphericity (χ^2^ = 4114.956, *p* < 0.001), indicating that the data were suitable for factor analysis. Only one common factor with an eigenvalue greater than 1 was extracted, which cumulatively accounted for 46.63% of the total variance. The factor loadings for each item were as follows: knowledge mastery (0.721), policy awareness (0.720), service evaluation (0.721), and behavioral confidence (0.555). Internal consistency reliability analysis showed that the overall Cronbach’s *α* coefficient for the composite measure was 0.609. The corrected item-total correlations (i.e., item-total correlation) for each item were: knowledge mastery (0.431), policy awareness (0.422), service evaluation (0.422), and behavioral confidence (0.296).

These results meet the basic reporting requirements for factor structure and internal consistency. The factor analysis supports that the four indicators share a common underlying dimension, providing preliminary validity evidence for their combination. At the same time, the *α* coefficient at the acceptable threshold and the variability in item-total correlations empirically reflect the conceptual heterogeneity of this measure: it integrates confidence in one’s own ability (behavioral confidence) with the cognition and evaluation of external environments and systems (knowledge, policy, service). Therefore, this composite score represents a multifaceted, domain-specific measurement of self-efficacy for blood donation, which incorporates not only perceived behavioral confidence but also the relevant knowledge and systemic trust necessary for the action. Based on social cognitive theory and the research objectives, it was still used as an operational measure of “self-efficacy” in subsequent analyses, with its psychometric properties fully considered in the discussion.

### 3.3 Univariate analysis of sociodemographic characteristics by blood donation behavior

The study included a total of 9,622 valid participants. The sociodemographic characteristics of the sample and the results of univariate analyses with blood donation behavior are summarized in Table 2. Among all surveyed participants, 78.2% (7,527/9,622) had no history of blood donation, 9.7% (937/9,622) were first-time donors, and 12.0% (1,158/9,622) were repeat donors.

**Table 2:** Sociodemographic characteristics and univariate analysis of blood donation behavior [*n* (%)].

	Non-blood donation	Donate blood once	Donate blood multiple times	χ^2^	*P* value
Gender					
Male	4,951 (65.8%)	614 (65.5%)	732 (63.2%)	2.921	0.232
Female	2,576 (34.2%)	323 (34.5%)	426 (36.8%)		
Age					
0–18	2005 (26.6%)	69 (7.4%)	39 (3.4%)	4325.534	*P* < 0.001
18–25	5,170 (68.7%)	564 (60.2%)	245 (21.2%)		
25–35	168 (2.2%)	149 (15.9%)	229 (19.8%)		
35–45	100 (1.3%)	95 (10.1%)	333 (28.8%)		
45–60	60 (0.8%)	59 (6.3%)	292 (25.2%)		
>60	24 (0.3%)	1 (0.1%)	19 (1.6%)		
Education level					
Bachelor’s degree or above	773 (10.3%)	257 (27.4%)	356 (30.7%)	1109.697	*P* < 0.001
Below junior high school	112 (1.5%)	56 (6.0%)	166 (14.3%)		
Junior college	4,443 (59.0%)	465 (49.6%)	363 (31.3%)		
Senior high school	2,197 (29.2%)	159 (17.0%)	272 (23.5%)		
Identity					
Urban resident	3,054 (40.6%)	548 (58.5%)	814 (70.3%)	423.244	*P* < 0.001
Rural resident	4,473 (59.4%)	389 (41.5%)	344 (29.7%)		
Occupation					
Service staff	22 (0.3%)	22 (2.3%)	66 (5.7%)	3897.845	*P* < 0.001
Individual business owner	29 (0.4%)	11 (1.2%)	50 (4.3%)		
Worker	25 (0.3%)	20 (2.1%)	95 (8.2%)		
Company clerk	49 (0.7%)	70 (7.5%)	173 (14.9%)		
Soldier	55 (0.7%)	12 (1.3%)	7 (0.6%)		
Farmer	87 (1.2%)	30 (3.2%)	81 (7.0%)		
Student	6,810 (90.5%)	532 (56.8%)	189 (16.3%)		
Government staff	163 (2.2%)	123 (13.1%)	288 (24.9%)		
Freelancer	26 (0.3%)	10 (1.1%)	18 (1.6%)		
Other	261 (3.5%)	107 (11.4%)	189 (16.3%)		

χ^2^ tests were used to compare the distribution of blood donation behaviors (non-donors, first-time donors, and repeat donors) across different demographic characteristics. The “0–18 years” age group was excluded from subsequent multivariate analyses as they did not meet the age requirement for blood donation.

Chi-square tests revealed statistically significant differences in the sociodemographic composition across non-donors, first-time donors, and repeat donors (Table 2). Detailed examination of column percentages highlighted distinct demographic profiles for each donor group. Gender distribution was balanced and did not differ significantly across the three groups (*p* = 0.232), with males consistently comprising approximately two-thirds of each group. A pronounced age-related shift was observed (*p* < 0.001). Younger individuals (18–25 years) constituted the majority of non-donors (68.7%) and first-time donors (60.2%), but their proportion dropped sharply among repeat donors (21.2%). Conversely, middle-aged adults (25–60 years), who represented less than 5% of non-donors, collectively formed the majority of repeat donors (73.8%). Education level also showed a significant gradient (*p* < 0.001). Individuals with a bachelor’s degree or higher were underrepresented among non-donors (10.3%) but were substantially overrepresented among both first-time (27.4%) and repeat donors (30.7%). A strong urban–rural disparity was evident (*p* < 0.001). Urban residents comprised 40.6% of non-donors, but this proportion increased to 58.5% among first-time donors and further to 70.3% among repeat donors. Occupational composition varied dramatically (*p* < 0.001). Students were the dominant group among non-donors (90.5%) and still formed a majority of first-time donors (56.8%), but they were a distinct minority among repeat donors (16.3%). In contrast, employed professionals such as government staff and company clerks, while minor components of the non-donor pool (2.2 and 0.7%, respectively), constituted substantial and increasing shares of donor groups, reaching 24.9 and 14.9% among repeat donors, respectively.

In summary, repeat donors were demographically distinct: they were predominantly middle-aged, urban, better-educated, and drawn from stable employment sectors, whereas non-donors were overwhelmingly young students.

### 3.4 Comparison and visualization of core psychological variables across different blood donation behavior groups

To examine the differences in altruistic factors and comprehensive self-efficacy (comprehensive readiness) across different blood donation behavior groups, a one-way analysis of variance (ANOVA) was conducted. Levene’s test indicated significant heterogeneity of variance for both altruistic factors score (*F* (2, 9,619) = 22.81, *p* < 0.001) and comprehensive self-efficacy score (*F* (2, 9,619) = 31.42, *p* < 0.001). Therefore, robust Welch’s ANOVA results were reported, and the Games-Howell post-hoc comparison method, which does not assume homogeneity of variance, was applied for pairwise comparisons.

Welch’s ANOVA revealed significant group differences in altruistic factors score, Welch’s *F* (2, 1814.04) = 47.95, *p* < 0.001. Group differences in comprehensive self-efficacy score were also highly significant, Welch’s *F* (2, 1818.12) = 1009.66, *p* < 0.001.

Specifically (see Table 3), post-hoc comparisons (Games-Howell) indicated: Altruistic factors score: Both first-time donors (M = 1.44) and repeat donors (M = 1.50) scored significantly higher than non-donors (M = 1.29) (all *p* < 0.001). However, the difference between first-time and repeat donors was not significant (*p* = 0.234). Comprehensive self-efficacy score: A clear gradient pattern emerged. Repeat donors had the highest scores (M = 2.40), significantly higher than both first-time donors (M = 2.14, *p* < 0 0.001) and non-donors (M = 1.76, *p* < 0.001). First-time donors also scored significantly higher than non-donors (*p* < 0.001).

**Table 3:** Comparison of altruistic factors and self-efficacy scores across different blood donation groups.

	Non-donors (*n* = 7,527)	First-time donors (*n* = 937)	Repeat donors (*n* = 1,158)	Welch’s F (df1, df2)	*P* value	Post-hoc comparisons
Altruistic factors score	1.29 ± 0.86	1.44 ± 0.79	1.50 ± 0.72	47.95 (2, 1814.04)	*P* < 0.001	2 ≈ 3 > 1
Self-efficacy score	1.76 ± 0.53	2.14 ± 0.46	2.40 ± 0.48	1009.66 (2, 1818.12)	*P* < 0.001	3 > 2 > 1

Data are presented as mean ± standard deviation. In the post-hoc comparisons column, 1 = non-donors, 2 = first-time donors, 3 = repeat donors. “≈” indicates non-significant difference, “>” indicates that the former is significantly higher than the latter (all comparisons based on Games-Howell test, *p* < 0.05).

**Figure 1:**
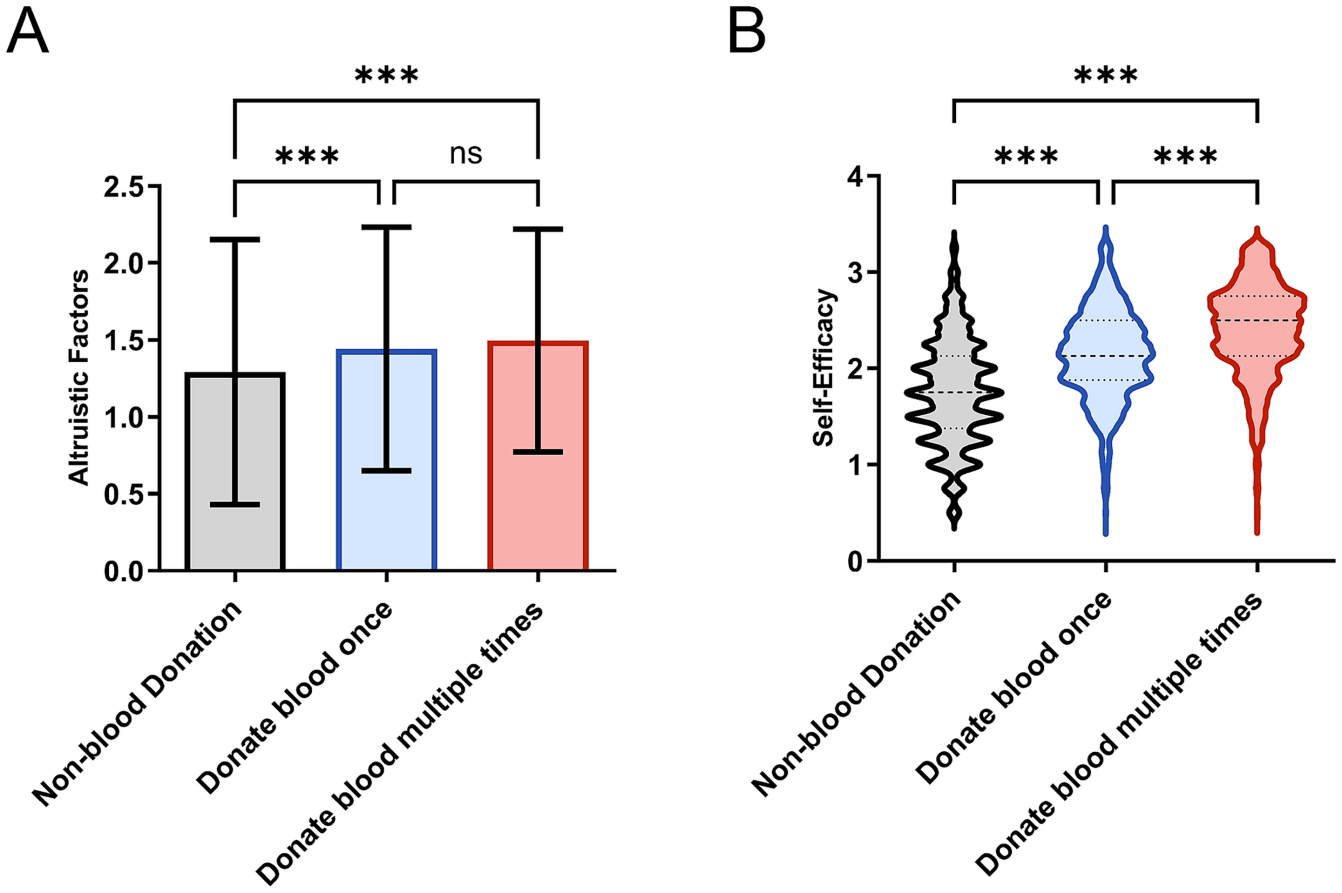
**(A)** Impact of altruistic factors on blood donation behavior, demonstrating a dose-dependent increase in donation rates with ascending altruistic factors scores. **(B)** Distribution of self-efficacy scores across different donor groups, showing significantly elevated median values and distinct distribution patterns in repeat donors compared to other groups. *** *p* < 0.001; ns denotes non-significant difference.

The above statistical differences were visually corroborated in Figure 1. Figure 1A illustrates the association between altruistic factors score and donation behavior: as altruistic factors score increases, the proportion of donors (particularly repeat donors) shows a marked upward trend, confirming a dose–response relationship. Figure 1B clearly displays the distributional differences in self-efficacy scores: the repeat donor group exhibits the highest and most concentrated score distribution, followed by the first-time donor group, with the non-donor group showing the lowest scores. This visual pattern is fully consistent with the statistical conclusions in Table 3, jointly indicating that higher altruistic motivation and stronger comprehensive self-efficacy are associated with more active and sustained donation behavior.

### 3.5 Multivariable logistic regression analysis of factors associated with blood donation behavior

Using “non-donation” as the reference category, a multivariable logistic regression analysis was performed with donation status (first-time donation and repeat donation) as the dependent variable, incorporating altruistic factors, comprehensive self-efficacy, and demographic variables. The results are presented in Table 4.

**Table 4:** Results of multivariable logistic regression analysis of factors associated with blood donation behavior (first-time vs. repeat donation).

Variable (reference)	Category	Regression coefficient (B)	*P* value	OR (95% CI)
Altruism (reference: low altruistic factors scores)	Medium altruistic factors scores	0.514	<0.001	1.673 (1.356–2.063)
	High altruistic factors scores	0.824	<0.001	2.281 (1.845–2.818)
	Very high altruistic factors scores	0.952	<0.001	2.592 (1.963–3.422)
Gender (reference: male)	Female	−0.459	<0.001	0.632 (0.547–0.729)
Age group (reference: 18–25 years)	26–35 years	1.164	<0.001	3.204 (2.466–4.161)
	36–45 years	2.151	<0.001	8.596 (6.058–12.199)
	46–55 years	2.734	<0.001	15.397 (10.693–22.172)
	56–60 years	3.054	<0.001	21.204 (14.233–31.591)
	Over 60 years	1.448	<0.001	4.255 (2.137–8.469)
Education level (reference: junior high school or below)	High school	−0.458	0.012	0.632 (0.443–0.903)
	Associate degree	−0.768	<0.001	0.464 (0.325–0.663)
	Bachelor’s degree or above	−0.369	0.053	0.691 (0.475–1.005)
Residence (reference: rural)	Urban	0.36	<0.001	1.433 (1.252–1.639)
Occupation (reference: other occupations)	Government staff	−0.616	0.088	0.540 (0.266–1.097)
	Enterprise employee	0.129	0.717	1.138 (0.566–2.289)
	Freelancer	0.275	0.382	1.316 (0.711–2.438)
	Worker	−1.74	<0.001	0.176 (0.080–0.387)
	Service industry	−0.777	0.014	0.460 (0.247–0.855)
	Farmer	−2.118	<0.001	0.120 (0.070–0.208)
	Self-employed	−0.467	0.106	0.627 (0.356–1.104)
	Student	−1.143	0.005	0.319 (0.143–0.709)
	Retired	−0.655	0.02	0.519 (0.299–0.903)

The analysis used “non-donation” as the reference group. Results show the effects of altruism level (reference: low level), self-efficacy (continuous variable), gender, age, education level, residential location, and occupation on donation behavior. OR, odds ratio; CI, confidence interval. Variable categorizations are detailed in the Methods section.

Altruistic Factors: Compared to the low-altruism group, the medium-, high-, and very high-altruism groups showed progressively increasing odds ratios (ORs) for blood donation behavior, at 1.673, 2.281, and 2.592, respectively (all *p* < 0.001), demonstrating a clear dose–response relationship. Comprehensive self-Efficacy: Higher comprehensive self-efficacy scores were significantly associated with increased likelihood of being a blood donor, particularly a repeat donor (*p* < 0.001).

Demographic Variables: Age significantly influenced donation behavior. Compared to the 18–25 years reference group, all older age groups showed progressively increased odds of donation, peaking in the 46–55 years group (OR = 15.397, *p* < 0.001). Females had significantly lower odds of donation than males (OR = 0.632, *p* < 0.001). Urban residents demonstrated significantly higher donation probability than rural residents (OR = 1.433, *p* < 0.001). Regarding occupation, workers, farmers, students, and retirees all showed significantly lower donation probabilities compared to the “other occupations” reference category (Table 4). The “other” occupational category (reference group) included homemakers, contract workers in large institutions, and stable professionals not covered by other groups. Its higher donation probability likely reflects the urban residence and stable social roles common among these subgroups, rather than a direct effect of occupational status per se.

### 3.6 Mediation analysis of comprehensive self-efficacy

In this study, altruistic factors were measured as a sum score (range: 0–3) derived from three core types of altruistic motivation for blood donation. Cognitive factors were assessed as a composite score covering four dimensions: knowledge level, information channels, publicity exposure, and knowledge need. Comprehensive self-efficacy was evaluated as a composite score comprising four dimensions: knowledge perception, behavioral confidence, policy awareness, and service evaluation. Detailed measurement procedures are provided in Section 2.2. The mediating role of comprehensive self-efficacy in the relationship between altruistic and cognitive factors with blood donation behavior was examined using the PROCESS v5.0 macro (Model 4). Two separate mediation analyses were conducted, with bootstrap sampling of 5,000 iterations for each. In the first model with altruistic factors as the independent variable, altruistic factors significantly predicted comprehensive self-efficacy (unstandardized *β* = 0.2286, *p* < 0.001). Comprehensive s elf-efficacy, in turn, significantly predicted donation behavior (unstandardized *β* = 1.4658, *p* < 0.001). The direct effect of altruistic factors on donation behavior was not significant (unstandardized *β* = 0.0037, *p* = 0.927). The indirect effect through comprehensive self-efficacy was 0.3351, with a 95% bootstrap confidence interval of [0.3009, 0.3715], indicating a significant indirect effect whereby comprehensive self-efficacy accounts for the observed association. In the second model with cognitive factors as the independent variable, cognitive factors significantly predicted comprehensive self-efficacy (unstandardized *β* = 0.0770, *p* < 0.001). Comprehensive self-efficacy significantly predicted donation behavior (unstandardized *β* = 1.5031, *p* < 0.001). The direct effect of cognitive factors on donation behavior was not significant (unstandardized *β* = −0.0091, *p* = 0.344). The indirect effect was 0.1157, with a 95% bootstrap confidence interval of [0.1040, 0.1275], likewise supporting a significant mediating role of comprehensive self-efficacy. However, we interpret this finding cautiously, as it may be influenced by the specific operationalization of variables and the cross-sectional nature of the study.

**Figure 2:**
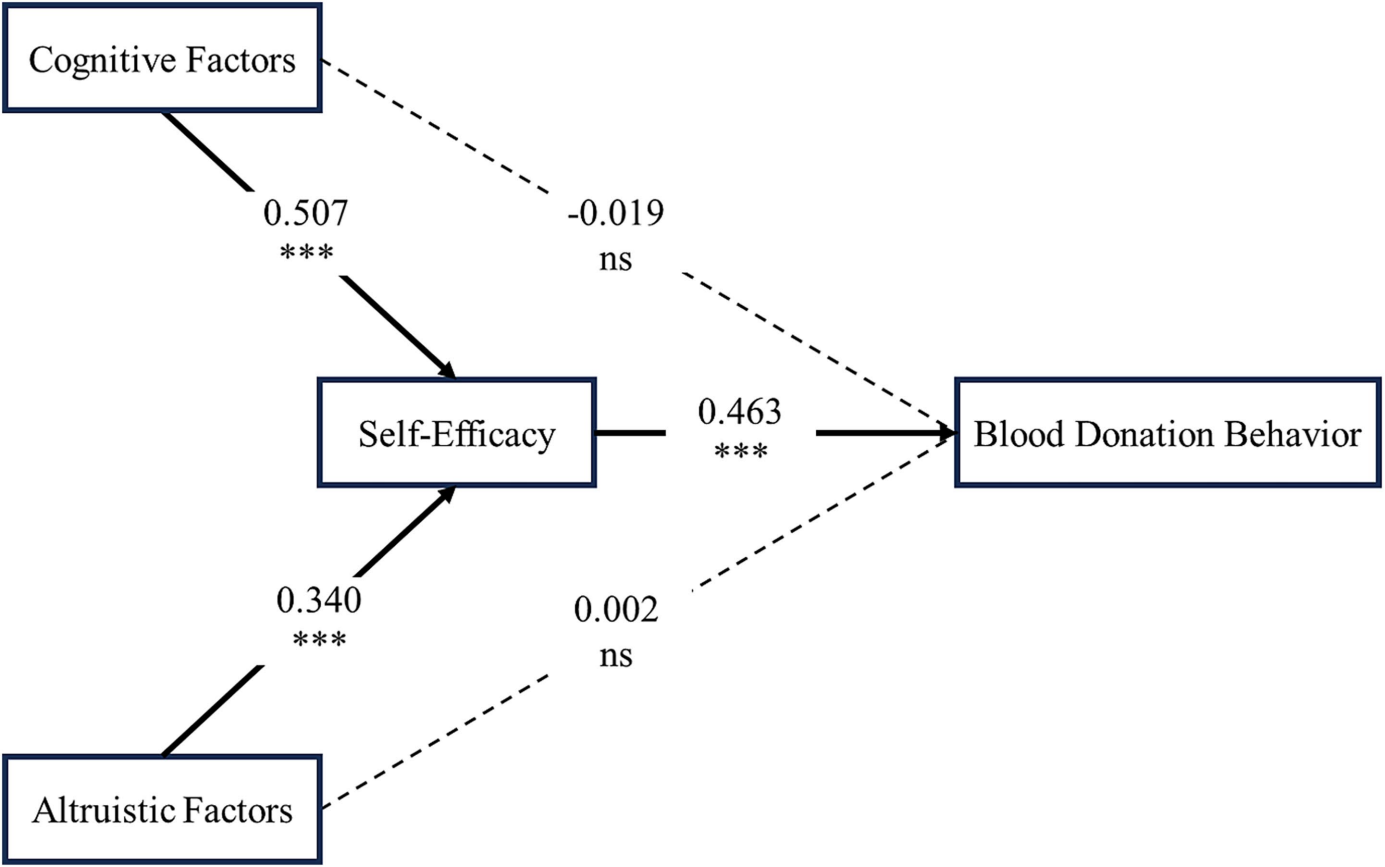
The mediation model illustrating the role of self-efficacy in the relationships between altruistic factors, cognitive factors, and blood donation behavior. *** *p* < 0.001; NS, non-significant. Solid arrows indicate significant paths; dashed arrows indicate non-significant paths.

For visual comparison, path coefficients were standardized. As shown in Figure 2, both altruistic factors (*β* = 0.340) and cognitive factors (*β* = 0.507) both positively predicted comprehensive self-efficacy, which in exerted a strong positive effect on blood donation behavior (*β* = 0.463). The direct effects of altruistic and cognitive factors on blood donation behavior were non-significant (*β* = 0.002 and −0.019, respectively). These results collectively support that comprehensive self-efficacy acts as a key mediator in the relationships between both altruistic/cognitive factors with blood donation in the tested model.

## 4 Discussion

Based on a large cross-sectional sample, this study investigated the role of altruistic factors and a multifaceted construct of comprehensive self-efficacy for blood donation in voluntary blood donation behavior. Key findings indicate that: (1) altruistic factors showed a dose–response relationship with donation behavior; (2) comprehensive self-efficacy was a strong independent predictor of donation; and (3) bootstrap mediation analysis revealed a statistical pattern consistent with full mediation, suggesting that comprehensive self-efficacy could account for the observed association between altruistic factors and blood donation behavior. Furthermore, significant demographic disparities were observed.

Altruistic factors showed a clear dose–response relationship with donation behavior, supporting their main role in motivating donation (9, 19, 29, 30). However, this motivation does not always translate into action, reflecting the well-documented “intention-behavior gap” among students. Despite strong altruistic ideals, students shows lower donation rates, likely due to structural and psychological barriers such as unfamiliarity with procedures, fear, or time constraints (25, 31). Interventions should go beyond altruistic appeals and include targeted support.

A pivotal finding was the statistical pattern consistent with full mediation: the relationship between altruistic factors and donation behavior can be explained by comprehensive self-efficacy. This suggests altruistic motivation may promote donation mainly by enhancing individuals’ perceived capability and preparedness for the donation process. These results align with social cognitive theory, while our broader operationalization of self-efficacy extends the traditional concept (13, 20, 21, 32). For structured healthcare behavior like blood donation, altruistic intention alone may be insufficient without adequate cognitive confidence and trust in the donation system. This pattern consistent with full mediation should be interpreted cautiously. Although consistent with our theoretical framework, the cross-sectional design limits causal inference, as the temporal order required for mediation cannot be confirmed. Additionally, the relatively brief measure of altruism may have reduced the chance of detecting direct effects. Longitudinal studies with more comprehensive measures are needed to verify this result.

Demographic factors (age, residence, occupation) were significant predictors of donation behavior, highlighting distinct donor characteristics and the need for targeted interventions. For students, low repeat donation may relate to insufficient confidence and procedural knowledge, supporting interventions such as graduated exposure programs and peer modeling. For rural residents, barriers mainly involve access and policy awareness, which could be addressed through mobile donation services and community education. Similarly, the higher donation probability in the “other” occupation group is interpretable given its composition, which includes urban-dwelling individuals with stable community ties or workplace-based recruitment access. This finding underscores the heterogeneity captured by occupational categories.

This study integrates altruism theory with an expanded self-efficacy framework, providing a “motivation-readiness-action” model of blood donation behavior whose core innovations are theoretical integration, indigenized construct expansion, and empirical validation based on a large-scale Chinese sample, which distinguishes it from existing models. This model expands traditional single-dimensional self-efficacy into a comprehensive blood donation readiness (integrating knowledge, confidence, policy awareness and service evaluation) that combines internal individual capabilities and external system trust, and integrates altruistic factors with self-efficacy to confirm the latter as the key mediator between altruistic motivation and blood donation behavior, theoretically explaining the intention-behavior gap in blood donation and offering a new unified analytical perspective for relevant psychological mechanism research. Practically, recruitment strategies should move beyond altruistic messages to actively build public self-efficacy to donate, including procedural knowledge, situational confidence and trust in the donation system.

In this study, the Cronbach’s *α* coefficient of the comprehensive self-efficacy scale was 0.609, slightly below the conventional threshold. This can be mainly attributed to the multidimensional nature of the scale, which covers four relatively independent yet interrelated domains: knowledge perception, behavioral confidence, policy awareness, and service evaluation. Differences in the focus of these subdomains tend to reduce the overall internal consistency to some extent. Nevertheless, the use of a composite total score remains justified and valuable in this study. First, the scale was developed based on the core construct of comprehensive psychological readiness for blood donation, and the composite total score provides a more holistic representation of individuals’ overall blood donation–related self-efficacy. Second, the total score significantly predicted blood donation behavior and showed a robust and significant mediating effect in the model, demonstrating sound empirical validity and explanatory power that meet the analytical objectives of this study. Future research may refine the scale items to further improve its internal consistency.

Several limitations should be noted. First, the cross-sectional design prevents definitive causal inferences about the mediation pathways; longitudinal or experimental designs are needed. Second, the sample was restricted to two cities in Henan Province, limiting generalizability. Third, self-reported donation behavior may be affected by recall or social desirability bias. Fourth, the composite self-efficacy scale had a Cronbach’s *α* of 0.609, near the lower acceptable range, requiring further validation and refinement. Fifth, the pattern consistent with full mediation may be influenced by our measurement and sample, and alternative mediators cannot be ruled out. Finally, altruistic factors was assessed by a single-item measure; future studies should use validated, multidimensional scales. Future studies should validate the psychosocial readiness construct in diverse populations and use longitudinal designs to confirm the proposed mediation model. Further studies are also needed to develop and test targeted interventions designed to enhance the specific components of readiness (e.g., knowledge workshops, service improvements to improve donation readiness and bridge the intention-behavior gap).

## 5 Conclusion

In conclusion, this cross-sectional study demonstrates that altruistic factors are associated with blood donation behavior in a dose–response manner, and that comprehensive self-efficacy serves as a key mediator in this relationship. Demographic characteristics are also related to donation behavior, with differing barriers across subgroups.

These findings support a “motivation–readiness–action” perspective and suggest that promoting blood donation requires both fostering altruistic values and improving public readiness related to donation. Enhancing knowledge, service experience, and trust may help translate altruistic willingness into actual donation behavior and contribute to a sustainable blood supply.

## Data Availability

The original contributions presented in the study are included in the article/supplementary material, further inquiries can be directed to the corresponding authors.
